# Ugonin inhibits chondrosarcoma metastasis through suppressing cathepsin V via promoting miR-4799-5p expression

**DOI:** 10.7150/ijbs.106827

**Published:** 2025-01-13

**Authors:** Nguyen Bao Tran, Ting-Kuo Chang, Nguyen Duong Phuong Chi, Kuan-Ying Lai, Hsien-Te Chen, Yi-Chin Fong, Chih-Chuang Liaw, Chih-Hsin Tang

**Affiliations:** 1 Graduate Institute of Biomedical Sciences, China Medical University, Taichung, Taiwan, 404.; 2 Department of Medicine, Mackay Medical College, New Taipei, Taiwan, 252.; 3 Division of Spine Surgery, Department of Orthopedic Surgery, MacKay Memorial Hospital, New Taipei, Taiwan, 104.; 4 Department of Chinese Pharmaceutical Science and Chinese Medicine Resources, China Medical University, Taichung, Taiwan, 404.; 5 Department of Marine Biotechnology and Resources, National Sun Yat-sen University, Kaohsiung, Taiwan, 80424.; 6 Department of Sports Medicine, College of Health Care, China Medical University, Taichung, Taiwan, 404.; 7 Department of Orthopedic Surgery, China Medical University Hospital, Taichung, Taiwan, 404.; 8 Department of Orthopedic Surgery, China Medical University Beigang Hospital, Taichung, Yunlin, Taiwan, 651.; 9 Graduate Institute of Natural Products, Kaohsiung Medical University, Kaohsiung, Taiwan, 807.; 10 Department of Pharmacology, School of Medicine, China Medical University, Taichung, Taiwan, 404333.; 11 Chinese Medicine Research Center, China Medical University, Taichung, Taiwan, 404.; 12 Department of Medical Laboratory Science and Biotechnology, College of Medical and Health Science, Asia University, Taichung, Taiwan, 41354.

**Keywords:** chondrosarcoma, metastasis, ugonin V, cathepsin V, miR-4799-5p

## Abstract

Chondrosarcoma is a rare type of bone cancer that develops in cartilage cells. In recent years, the incidence of chondrosarcomas has steadily increased worldwide. During the advanced stages, chondrosarcoma carries a significant risk of metastasis and exhibits resistance to both chemotherapy and radiation therapy. Hence, the development of potent treatments for chondrosarcoma is an urgent requirement. Ugonin V is a flavonoid compound that has been extracted from the plant *Helminthostachys zeylanica* (L.) Hook. This study examined the molecular therapeutic effects of ugonin V on chondrosarcoma metastasis. Analysis of the GSE30835 dataset, which consists of chondrosarcoma tissues and normal cartilage, revealed significant upregulation of three cathepsin proteases in chondrosarcoma, namely cathepsin (CTS) A, L, and V. Notably, ugonin V specifically suppressed cathepsin V mRNA expression. We also found that ugonin V strongly inhibits chondrosarcoma cell motility by regulating CTSV expression. In addition, through miRNA sequencing, we observed that ugonin V targets CTSV via miR-4799-5p to effectively suppress chondrosarcoma cell migration and invasion. Our *in vitro* and *in vivo* studies provide an initial investigation of the involvement of cathepsin V and miR-4799-5p in chondrosarcoma metastasis after ugonin V treatmen

## Introduction

Chondrosarcoma is a rare primary bone cancer derived from cartilage cells. In recent years, there has been a general upward trend in the global incidence of chondrosarcomas 1. Tumor grade, metastasis, age, and location are important factors that significantly affect the overall survival of patients with this disease 2. Advanced-grade chondrosarcomas with high cellularity are associated with a high metastasis rate and poor prognosis 3-5. Chondrosarcomas are typically unresponsive to chemotherapy and radiotherapy; therefore, surgery is the primary treatment option. Patients with high-grade chondrosarcomas are at risk of experiencing both local recurrence and distant metastases following surgical removal 6-8. Therefore, many efforts have been made to increase the therapeutic effects on chondrosarcoma metastasis, especially by exploring molecular targets 8.

As of 2019, approximately 25% of new anticancer medications were derived from natural sources 9*.* Observations have shown that natural products can simultaneously affect multiple oncogenic signaling pathways by altering the activity or expression of their molecular targets. Diverse natural extracts affect numerous pathways, including apoptotic cell death, cell proliferation, migration, invasion, angiogenesis, and metastasis 10*.* There have been numerous reports on natural products with molecules targeting effects that inhibit cancer progression, such as curcumin 11, quercetin 12, and apigenin 13. Ugonins are a group of flavonoids extracted from *Helminthostachys zeylanica* (L.) Hook. (HZ) 14. Recently, several ugonin compounds with antitumor properties have been identified 15-18. Specifically, a previous study documented that ugonin P inhibited A549 and CL1-5 cell migration and invasion by regulating DPP4 expression 15. In addition, Yamauchi *et al.* demonstrated the inhibitory effect of ugonin K on extracellular melanogenesis 18. To date, only one study has documented the bioactivity of ugonin V in chondrosarcoma metastasis 17. Additional studies are required to determine the role of ugonin V in the treatment of chondrosarcoma.

The cathepsin family is a class of proteases initially discovered as intracellular peptide hydrolases, although some cathepsins also perform functions outside cells. The cathepsin family comprises cathepsin A, B, C, D, E, F, G, H, L, K, O, S, V, and W, as reported by Tan *et al.* in 2013 19. Cysteine proteases, which belong to the largest class of the cathepsin family, are expressed on the cell surface and released into the extracellular space. At this location, they can break down elements of the extracellular matrix (ECM) to facilitate the invasion of cancer cells, which is the initial step of metastasis 20*.* Cathepsin V (CTSV) is a cysteine protease associated with tumor progression and is correlated with poor prognosis in liver, colon, and breast cancers 21-23. However, the role of cathepsin V in the progression of chondrosarcoma remains unclear. Hence, it is essential to further investigate the correlation between cathepsin V levels and chondrosarcoma.

Several dysregulated microRNAs (miRNAs) specific to sarcomas have recently been discovered 24. These biomarkers are regarded as promising for diagnosing and predicting patient outcomes and are linked to tumor progression by regulating cell division, cell mobility, programmed cell death, and angiogenesis 25. In chondrosarcomas, different miRNAs alter expression levels in cell lines and tumor samples 26. In 2020, Parafioriti *et al.* identified 17 critical miRNAs that regulated pathways involved in the formation and growth of chondrosarcoma 27. Several studies have demonstrated that promoting miR-342-5p and miR-520f-3p expression suppresses lung cancer and chondrosarcoma cell movement 28, 29. Much evidence exists regarding the anticancer effects of natural products, especially polyphenols, through the modulation of miRNA expression 30. As polyphenol compounds, both genistein and curcumin have been shown to upregulate miR-34a expression, which contributes to the inhibition of head and neck cancer 31 and prostate cancer 32. In bone cancer research, resveratrol has been shown to exert anticancer activity by inducing levels of miR-139-5p expression 33. Our study reveals that miR-4799-5p acts as a mediator of ugonin V to inhibit CTSV expression and chondrosarcoma progression *in vitro* and *in vivo.* We investigated the inhibitory effects of miR-4799-5p on CTSV expression and chondrosarcoma cell migration and invasion. This study aimed to investigate the effect of ugonin V on chondrosarcoma metastasis through the inhibition of CTSV expression mediated by miR-4799-5p.

## Material and methods

### Material

Ugonin V was isolated by Dr. Chih-Chuang Liaw (National Sun Yat-sen University, Taiwan) following a protocol reported in a 2010 publication 14. MTT ((3-(4,5-dimethylthiazol-2-yl)-2,5-diphenyltetrazolium bromide) buffer and β-Actin antibody were bought from Sigma-Aldrich (St. Louis, MO, USA). The Novolink Polymer Detection Systems kit was purchased from Leica Biosystems (St. Gallen, Switzerland). Cathepsin V antibody was purchased from GeneTex (Hsinchu City, Taiwan).

### Cell culture

The grade 2 mutant IDH2 SW1353 cell line was purchased from ATCC (Manassas, VA, USA) and the grade 2 mutant IDH1 JJ012 cell line was a gift from Dr. Sean P. Scully (University of Miami School of Medicine in Miami, FL, USA). These cell lines were cultured as described previously 34.

### Clinical chondrosarcoma tissues and normal cartilage

Tumor tissues and normal cartilage were obtained from patients diagnosed with chondrosarcoma and osteoarthritis, respectively. The procedures were conducted at the China Medical University Hospital. The study was conducted in accordance with the Declaration of Helsinki and was approved by the Institutional Review Board of China Medical University Hospital. Chondrosarcoma tumors and cartilage were stored at a temperature of -80°C until RNA extraction.

### MTT assay

JJ012 and SW1353 cells were seeded in a 96-well culture plate (5 × 10^3^ cells/well). After 24 h of culture, ugonin V with different concentrations (1 μM, 3 μM, 10 μM) was added and incubated for 24 and 48 h. The subsequent protocol followed that described previously 15.

### Migration assay

A quantity of 2.5 × 10^3^ cells per well was utilized to perform the migration assay using a 48-well Micro Chemotaxis Chamber (Neuroprobe, Gaithersburg, MD, USA) following our previous report 17.

### Invasion assay

Invasion assays were performed using an 8-µm pore-size Corning Costar Transwell chamber (St. Louis, MO, USA). Approximately 1 × 10^5^ cells were added to the upper chamber, which was coated with a thin layer of matrix gel. The cells were incubated in a 10% FBS medium with or without ugonin V for 24 h. Following the incubation period, the number of migrating cells was assessed according to the protocol outlined in our previous publication 35.

### mRNA and miRNA analysis

Total RNA was extracted from chondrosarcoma tumors or cell lysates using a TRIzol kit (MDBio). Transcripts were quantified using real-time quantitative polymerase chain reaction (qPCR) with SYBR Green (Applied Biosystems). The primer sequences used for RT-qPCR were as follows: CTSV forward, AAAGCAGTCGCAACTGTGG and reverse, GACGAGCCAATACTTGCTGTT; CTSA forward, TGGTCTACTTTGCCTACTACCAT and reverse, CACACGGGGCATAGAGATTG; CTSL forward, AAACTGGGAGGCTTATCTCACT and reverse, GCATAATCCATTAGGCCACCAT; GAPDH forward, ACCACAGTCCATGCCATCAC and reverse, TCCACCACCCTGTTGCTGTA. The cDNA for miRNA analysis was synthesized using the Mir-X™ miRNA First-Strand Synthesis kit from Clontech. The miR primers used were as follows miR-892b: CACTGGCTCCTTTCTGGGTAGA; miR-4799-5p, ATCTAAATGCAGCATGCCAGTC; miR-377-5p, AGAGGTTGCCCTTGGTGAATTC; miR-892a, CACTGTGTCCTTTCTGCGTAG. The mRNA levels and miRNA expression were assessed using the StepOnePlus sequence detection system (Applied Biosystems) 29.

### Bioinformatic analysis

High-quality total RNA samples from the control and treatment groups of JJ012 cells were used for miRNA sequencing analysis (GSE279984). The experimental workflow was performed by Anzenta Life Science Company (Burlington, MA, USA). One microgram of total RNA was used for library preparation. Prior to analysis, the raw reads were subjected to quality control, which included the removal of contamination and adapter sequences. Statistical analysis of the lengths and counts of the filtered reads, as well as data volume assessment, was performed to ensure data quality. Adapter sequences were removed using Trimmomatic (v0.30) to obtain clean data, and duplicate sequences were removed during the quality control phase. During the filtering process, statistical analysis of the lengths and counts of the filtered reads was conducted to evaluate data quality and ensure data completeness. Quality control was executed on the raw data by eliminating contaminants and adapter sequences, followed by statistical analysis of the filtered data to ascertain its quality. The filtered reads were aligned to the miRbase (V22) database, which consists of known miRNA sequences, followed by microRNA annotation. Additionally, the filtered reads were aligned to the Rfam (V14.1) database to study the distribution of non-coding RNAs. These steps were conducted using specific bioinformatics tools. During the data processing phase, analyses on miRNA differential expression, clustering, and target gene prediction were performed, helping to validate the accuracy of our assembly and annotation results.

Datasets representing miRNAs with altered expression profiles derived from miRNA sequencing were imported into the “MicroRNA Target Filter” of QIAGEN Ingenuity Pathway Analysis software (IPA) (Hilden, Germany) for further exploration. Analysis of miRNA expression changes in IPA identifies target mRNAs, common pathways and biological functions associated with different diseases.

The GSE30835 dataset from the Gene Expression Omnibus (GEO) database summarized the expression levels of cathepsin family proteases in cartilage and chondrosarcoma tissues. The GSE184118 dataset provides single-cell RNA sequencing from chondrosarcomas and a benign. The expression of CTSV is analyzed and visualized by Loupe Browser software.

### Transfection

Lipofectamine 2000 (Thermo Fisher Scientific Inc., IL, USA) was used to transfect 1 μg/μl of the control pcDNA3.1(+) plasmid or CTSV overexpressed plasmid (MD Bio Inc., Montgomery County, MD, USA) into the JJ012 and SW1353 cell lines to establish overexpression cell lines (JJ012/CTSV and SW1353/CTSV). After 24 h in cell culture conditions, 200 μg/mL of G418 (Geneticin) (Life Technologies) was used to select stable transfectant cells. Stable clone cell lines were selected as previously described 29.

The miR-4799-5p negative control and inhibitor (Allbio Science Incorporate, Taichung, Taiwan) have a sequence (5'-3') as follows: miR-4799-5p negative control (NC; CAGUACUUUUGUGUAGUACAA) and miR-4799-5p inhibitor (GACUGGCAUGCUGCAUUUAGAU). Chondrosarcoma cells (JJ012, SW1353) were transfected using Lipofectamine 2000 in a 6-well plate with 10 nM of the miR-4799-5p inhibitor or negative control under the culture conditions. Following transfection for 24 h, the serum-free medium was replaced with fresh culture medium, with or without ugonin V (10 μM), and incubated for an additional 24 h.

### Western blot

Chondrosarcoma cells were treated with ugonin V for 24 h. Total protein was extracted using RIPA lysis buffer and quantified using the BCA Protein Assay Kit (Thermo Fisher Scientific Inc., IL, USA). After separating the resolved proteins using SDS-PAGE, the gels were transferred onto polyvinylidene difluoride (PVDF) membranes. The membranes were blocked in a solution of 5% nonfat milk for 1 h at room temperature. Subsequently, the primary antibodies CTSV (1:2000) and β-actin (1:5000) were administered and left to incubate for 1 h at room temperature or overnight in the refrigerator at 4^o^C. The membranes were then incubated with a secondary antibody (1:2000) for 1 h. Immunoreactive bands were detected using an enhanced chemiluminescence reagent (ECL) from Merck Millipore (Burlington, MA, USA). The bands were observed using the Invitrogen iBright Imaging Systems (Thermo Fisher Scientific Inc., Rockford, IL, USA).

### Immunohistochemistry (IHC) staining

The OS805a tissue array was purchased from US Biomax (Rockville, MD, USA). The section was deparaffined and blocked according to our protocol in the previous study 29. Sections were deparaffinized in xylene and rehydrated in a series of washes with decreasing concentrations of ethanol. After citrate-based antigen retrieval and neutralizing endogenous peroxidase steps, the primary antibody (CTSV with a ratio of 1:100) was applied and incubated overnight in a 4^o^C refrigerator. Next, the slides were incubated with the secondary antibody for 30 min at room temperature. Then, 3,3'-diaminobenzidine (DAB) staining was performed, followed by hematoxylin staining, drying, and mounting. The expression of the CTSV protein was evaluated following the assessment score described in a previous study 17*.*

### Chondrosarcoma metastasis mouse model

The Institutional Animal Care and Use Committee authorized the animal protocols and procedures (CMUIACUC-2022-243-3). Four-week-old male BALB/c nude mice (15-18 grams per one) were purchased from the National Laboratory Animal Center (Taipei, Taiwan) and housed in the China Medical University Animal Center's animal facility. Mice were maintained in controlled settings (22±1˚C, 50±10% humidity, 12 h light/dark cycle) with daily food and water (normal diet). When mice acclimated to the environment, body weight was measured every week. One week later, 1 × 10^6^ luciferase reporter plasmid-transfected stable clone JJ012 (JJ012-luc) cells, which were suspended in 100 μl of DMEM serum-free, were injected intravenously into the lateral tail vein. The injected mice were randomly divided into three groups (n = 6 in each group), which were assigned to treatment with vehicle (phosphate-buffered saline (PBS)), ugonin V 5 mg/kg (low dose) or ugonin V 15 mg/kg (high dose). After 3 days of JJ012-luc cell injection, ugonin V (diluted in autoclaved PBS) was given intraperitoneal (IP) thrice weekly for a period of four weeks. Lung metastasis was assessed every week using a Xenogen IVIS Imaging System 200 (PerkinElmer, Waltham, MA, USA) by pre-injected with D-Luciferin, Potassium Salt (GoldBio LUCK, St. Louis MO, USA). The mice were maintained under anaesthesia with 1.5% isoflurane during the experiment. At the end of the experiment, after euthanasia with CO_2_ and confirmation of the animals' motionlessness, lack of respiration or pulse, and dilated pupils, death was confirmed after a further 2-3 min of observation. Immediately afterwards, approximately 0.5 ml of blood was collected from each group of mice via cardiac puncture and lung tissues were harvested for further experiments.

### Statistical analysis

Quantified data were analyzed using GraphPad Prism 10 software. Statistical significance was determined using the Student's *t*-test to compare two unpaired groups. To compare multiple groups, one-way ANOVA followed by Tukey's post-hoc test was used to analyze significant differences. Data are presented as mean ± standard deviation (SD) from at least three independent experiments. *p* < 0.05 indicates a statistically significant difference.

## Results

### Ugonin V effectively inhibits chondrosarcoma cell migration and invasion by reducing CTSV

**Figure 1:**
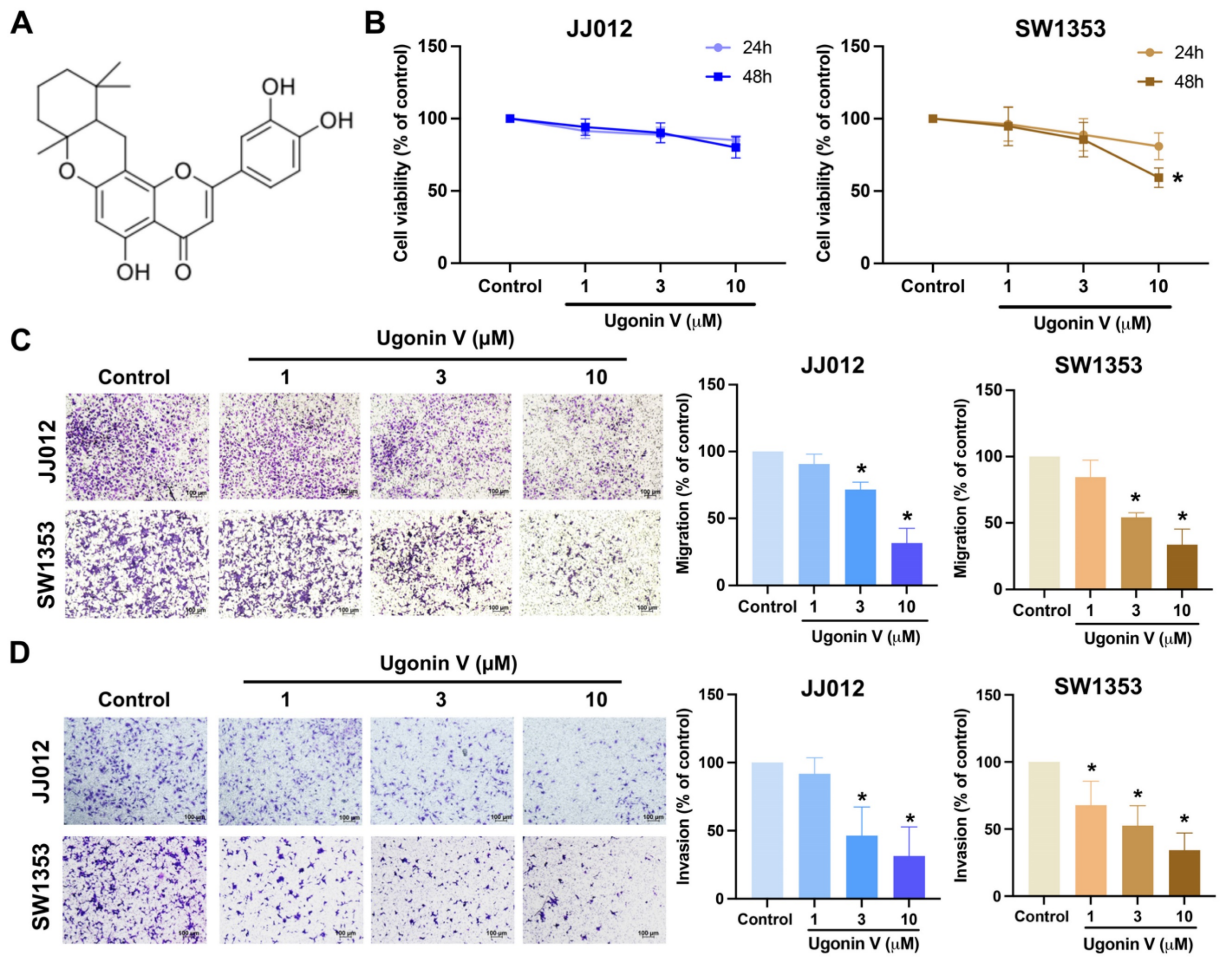
** Ugonin V inhibits chondrosarcoma cell migration and invasion in a concentration-dependent manner.** (A) The chemical structure of ugonin V. (B) JJ012 and SW1353 cells were treated with different concentrations of ugonin V (1 μM, 3 μM, and 10 μM) for 24 and 48 h, MTT assay was used to evaluate the cytotoxicity effect. (C&D) Migration and invasion assay determined the migration and invasion ability of JJ012 and SW1353 cells after 24 h incubated in different concentrations of ugonin V (1 μM, 3 μM, 10 μM). *p < 0.05.

Ugonin V is a flavone extracted from HZ (Fig. 1A). In the previous study, ugonin V showed noncytotoxic effects on chondrosarcoma cells after 24 h of treatment 17. In this study, we evaluated the effect of ugonin V over a longer period and found a significant decrease in SW1353 cells after 48 h of treatment (Fig. 1B). We then continued to investigate the effect of ugonin V in chondrosarcoma cell migration and invasion under 24 h treated with ugonin V in varying concentrations (1 μM, 3 μM, 10 μM). The results in Fig. 1C and D showed the notable inhibitory effect of ugonin V on JJ012 and SW1353 cell migration and invasion in a concentration-dependent manner.

**Figure 2:**
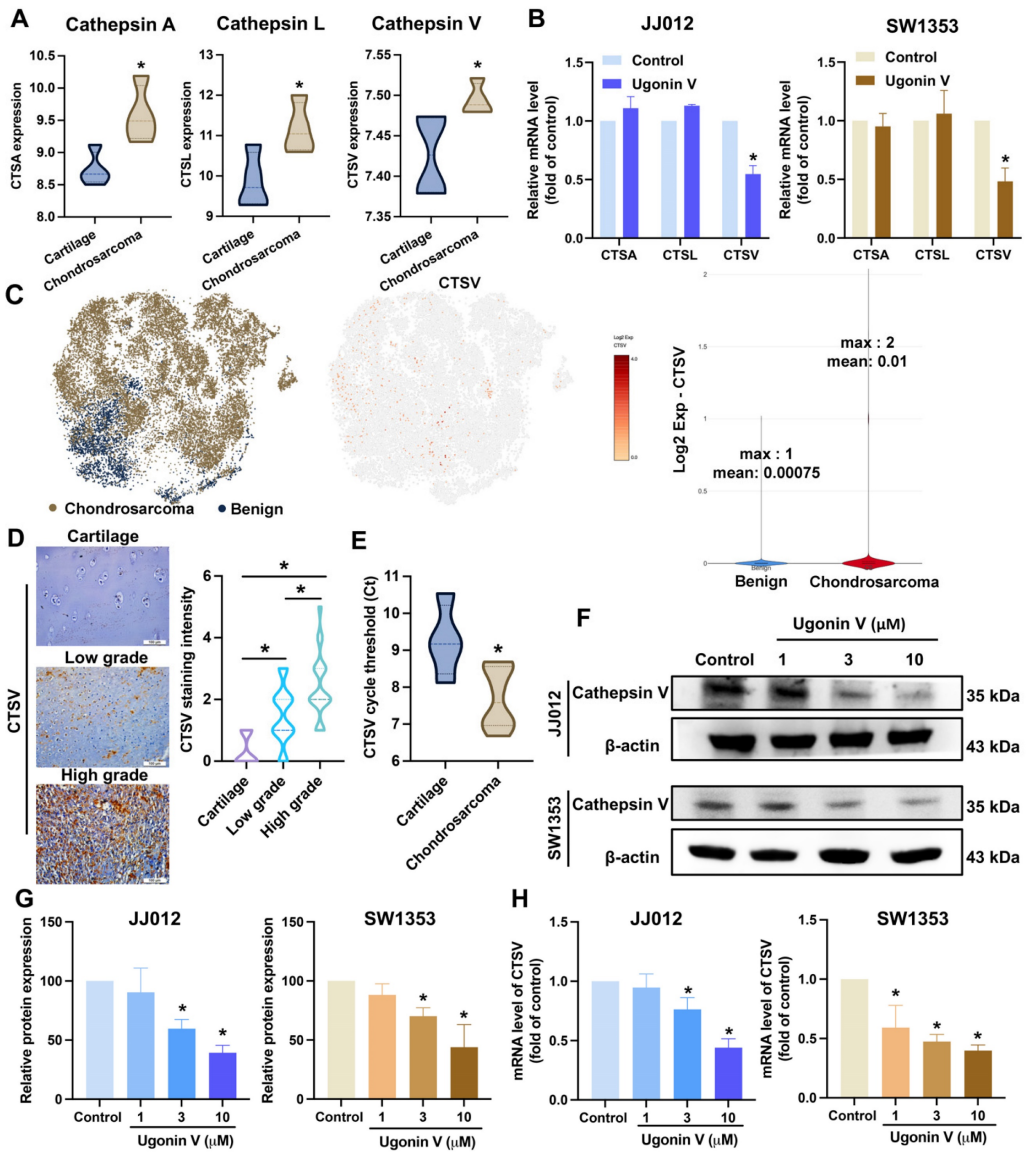
** Ugonin V effectively inhibits CTSV expression.** (A) The GSE30835 dataset was used to summarize the expression of the cathepsin family. Histogram of three proteases significant increase in chondrosarcomas, cathepsin A, cathepsin L, and cathepsin V. (B) The mRNA levels expressions of cathepsin A, cathepsin L and cathepsin V were assessed by qPCR after 24 h of incubating with ugonin V (10 μM). (C) The t-SNE and violin plots illustrate the expression of CTSV across patients. (D) Human tissue array OS805a was stained with CTSV antibody. (E) CTSV mRNA expression levels in patients' tumors were analyzed by threshold cycle (Ct) using qPCR. (F-H) CTSV protein expression and mRNA levels were assessed using western blotting and qPCR analysis after 24 h incubation with different concentrations of ugonin V (1 μM, 3 μM, 10 μM). *p < 0.05.

The cathepsin family was investigated in several reports for their essential role in cancer metastasis 19. We analyzed a chondrosarcoma dataset, GSE30835, from the GEO database to evaluate the expression of nine cathepsins (cathepsin A, B, C, D, E, L, S, V, and Z) and found that three cathepsins, A, L, and V, showed notably higher expression in chondrosarcoma tissues than in normal cartilage (Fig. 2A). However, when the JJ012 and SW1353 cells were treated with ugonin V, only CTSV mRNA expression was significantly suppressed (Fig. 2B). Single-cell RNA sequencing data analysis from GSE184118 indicated that CTSV expression was observed to be activated in conventional central chondrosarcoma patients (Fig. 2C). In addition, IHC staining results shown in Fig. 2D indicated significantly higher CTSV expression in chondrosarcoma sections compared to that in cartilage. Interestingly, CTSV expression was significantly higher in high-grade chondrosarcomas than in low-grade chondrosarcomas. We then examined the mRNA expression levels of CTSV in the tissues of patients with chondrosarcoma and noncancerous cartilage from osteoarthritis (OA) patients. The results in Fig. 2E show higher CTSV mRNA expression in chondrosarcoma tissues than in cartilage tissues. Collectively, these findings provide evidence of the remarkable expression of CTSV in patients with chondrosarcoma.

**Figure 3:**
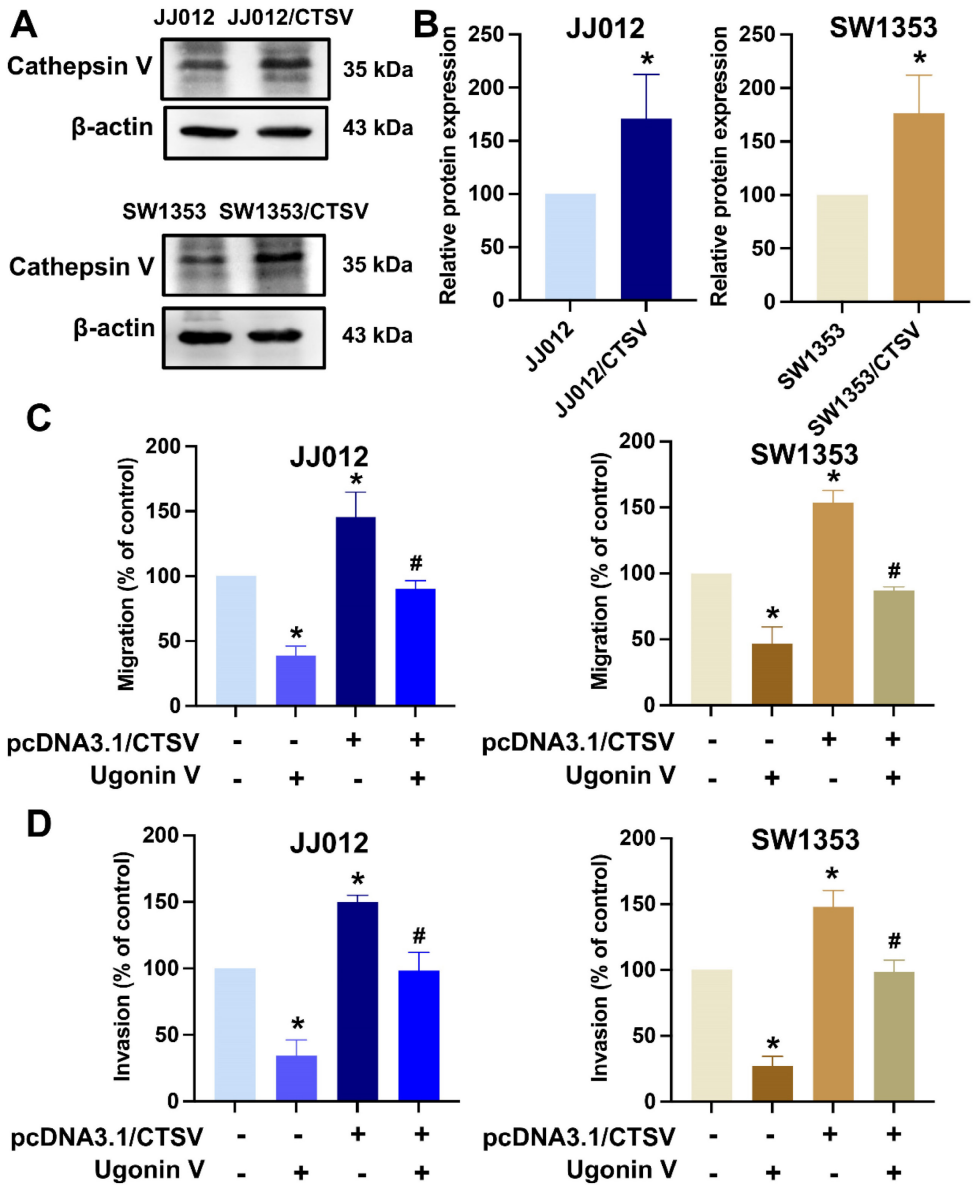
** Over-expression of CTSV reverses ugonin V effect in chondrosarcoma cell migration and invasion.** (A&B) Western blot analysis of CTSV expression in JJ012 and SW1353 after pcDNA3.1(+)/CTSV vector and empty vector were transfected. (C&D) Parental and CTSV-overexpressing JJ012 and SW1353 cell lines were treated with 10 μM ugonin V for 24 h, cell motility was respectively analyzed by migration and invasion assays. *p < 0.05 compared to control group; *^#^p* < 0.05 compared to the ugonin V-treated group.

Next, chondrosarcoma cells were treated with ugonin V to examine its effects on CTSV expression. Western blotting and qPCR analyses revealed a concentration-dependent decrease in CTSV expression (Fig. 2F-H). Moreover, when we overexpressed CTSV in JJ012 and SW1353 cells, the migration and invasion abilities of these cell lines were significantly enhanced, and the effect of ugonin V was reversed (Fig. 3). Therefore, ugonin V demonstrates an inhibitory effect on the regulation of chondrosarcoma cell migration and invasion via CTSV suppression.

### Ugonin V restricts chondrosarcoma cell migration and invasion by suppressing CTSV synthesis via inducing miR-4799-5p expression

**Figure 4:**
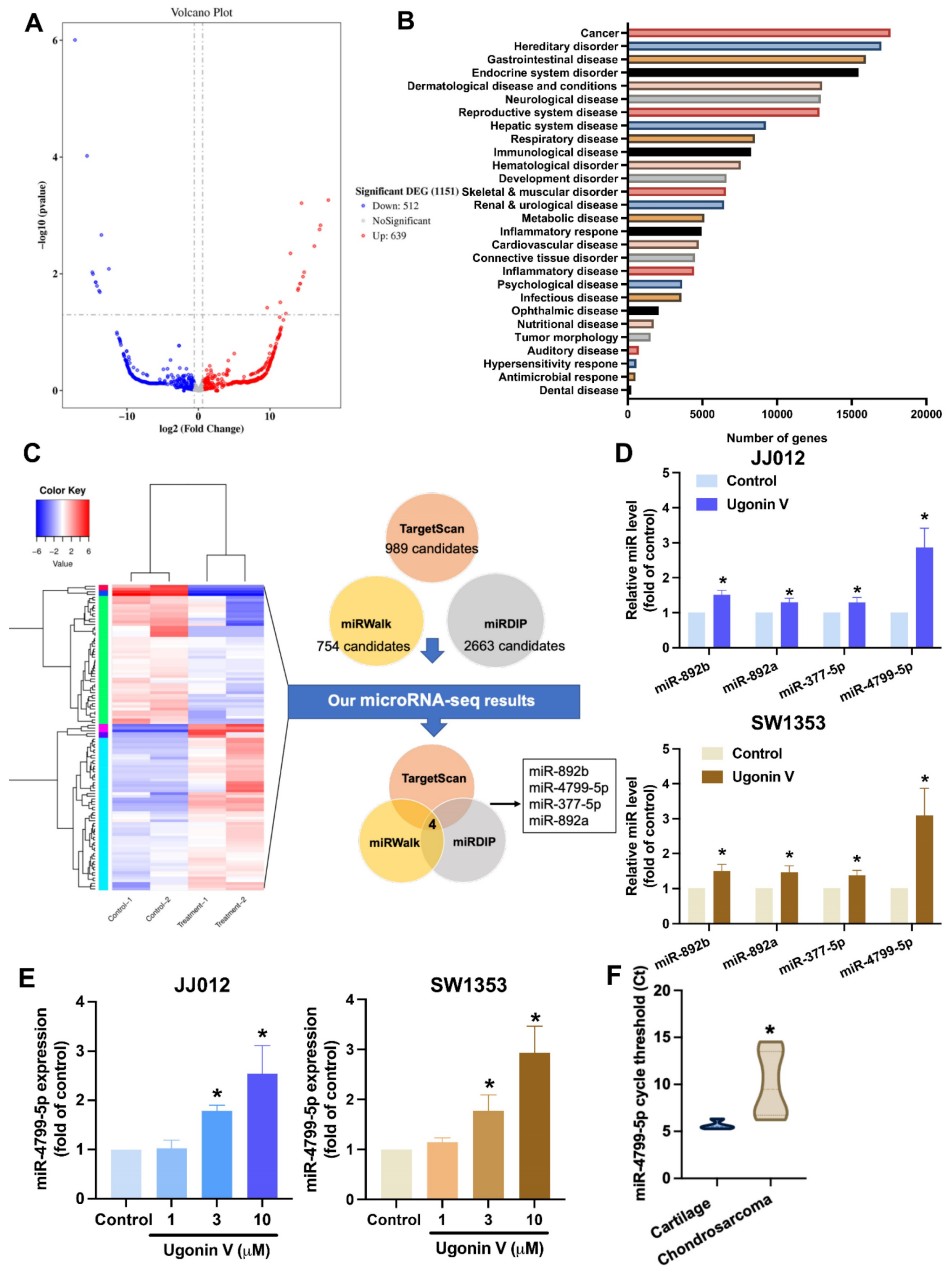
** Ugonin V promotes miR-4799-5p synthesis.** (A) Volcano plot of differentiated miRNAs in JJ012 cells after 24 h incubating with ugonin V (10 μM). (B) Histogram of diseases enriched from target genes of significantly differentiated miRNAs analyzed by IPA. (C) The miRNAs that predicted targeting CTSV by database analysis, Targetscan, and miRwalk, miRDIP, were merged with miRNA sequencing results in chondrosarcoma cell (JJ012) treated with or without ugonin V (10 μM). (D) The expressions of miR-892b, miR-892a, miR-377-5p, and miR-4799-5p were qualified by qPCR after 24 h of ugonin V treatment (10 µM). (E) The expression of miR-4799-5p was qualified by qPCR after 24 h of ugonin V dose-dependent treatment (1 µM, 3 µM, 10 µM). (F) MiR-4799-5p expressions in the patient's tumor were analyzed by threshold cycle (Ct) using qPCR. *p < 0.05. IPA, Ingenuity Pathway Analysis.

The roles of miRNAs in cancer metastasis have been reported in several previous studies 36, 37. We hypothesized that ugonin V controls the expression of CTSV by regulating the expression of miRNA. The miRNA sequencing analysis was performed to examine the differential expression of miRNAs in the JJ012 cell line treated with 10 μM ugonin V. We got 1151 differentiated miRNAs (512 downregulated miRNAs and 639 upregulated miRNAs), including well-known and novel miRNAs (Fig. 4A). When we used IPA to predict the well-known miRNAs, most target mRNAs were related to cancer (Fig. 4B). We investigated the regulation of CTSV expression by ugonin V mediated by certain miRNAs. Three publicly available miRNA databases (TargetScan, miRWalk, and miRDIP) predicting miRNAs that target CTSV were merged with the miRNA sequencing data to figure out the potential candidates. After filtering, four candidates targeting CTSV were identified (Fig. 4C). Subsequently, to validate the miRNA sequencing results, qPCR was performed on JJ012 and SW1353 cells to detect the mRNA expression of these candidates in the control and treatment groups. MiR-4799-5p showed an outstanding high expression after ugonin V treatment and a significant increase in a dose-dependent manner in both JJ012 and SW1353 cells (Fig. 4D and E). Interestingly, when we detected the expression of miR-4799-5p in chondrosarcoma tissues and the cartilage of noncancer patients, the Ct values of miR-4799-5p were significantly higher, indicating a lower expression of miR-4799-5p in chondrosarcoma (Fig. 4F).

**Figure 5:**
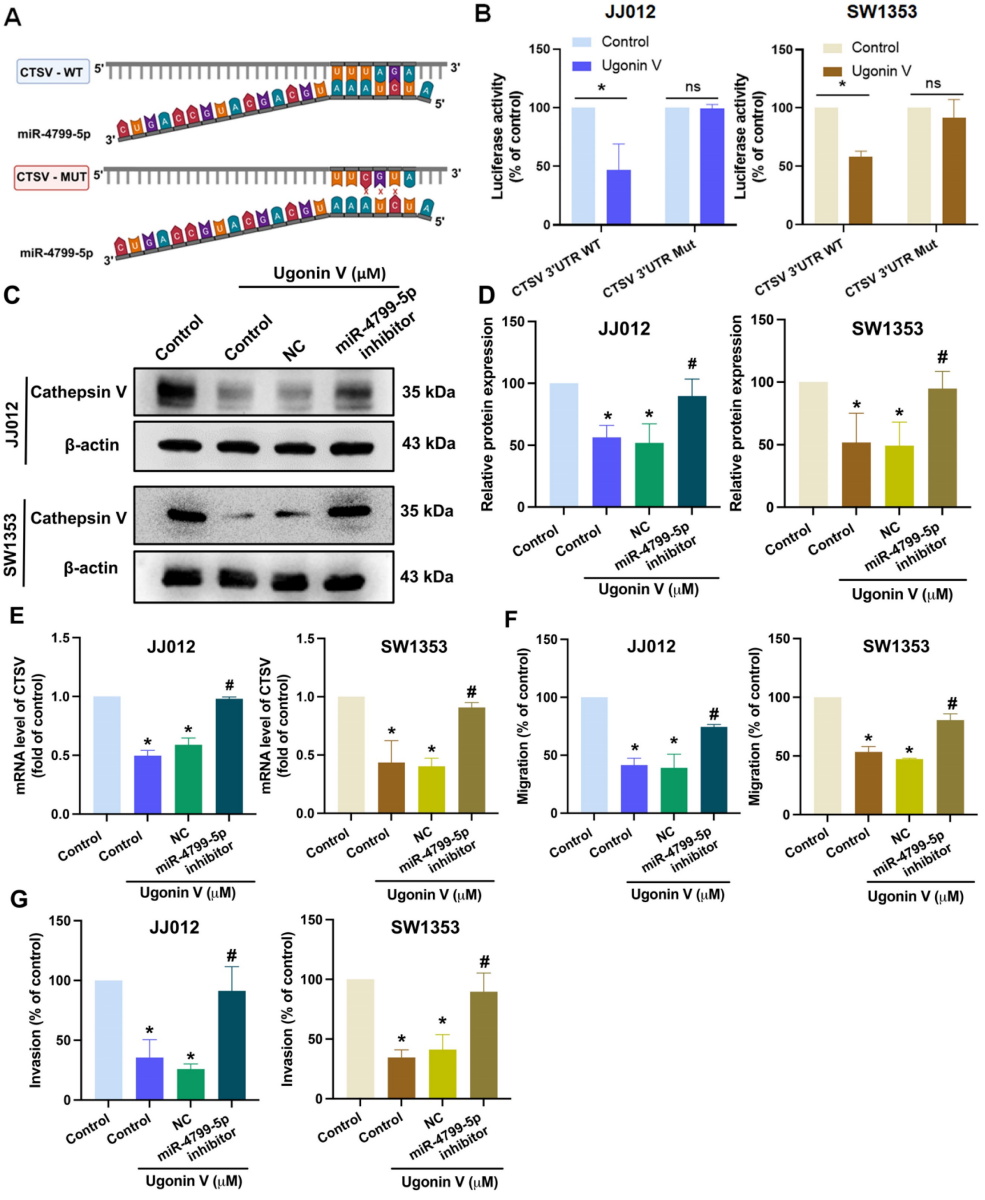
** MiR-4799-5p directly binds to CTSV 3'-UTR to reduce CTSV synthesis and suppress chondrosarcoma cell migration and invasion.** (A) The graphic illustrates the binding site of miR-4799-5p on CTSV 3'-UTR predicted by the TargetScan database. (B) Relative luciferase activities of chondrosarcoma cells transfected with a CTSV 3'-UTR luciferase reporter vector, after 24 h of ugonin V treatment. JJ012 and SW1353 cell lines were transfected with a miR-4799-5p inhibitor and negative control, followed by 24 h of ugonin V treatment (10 μM). (C-E) Western blotting and qPCR analysis were used to determine CTSV expression. (F&G) Chondrosarcoma cell motility was analyzed by migration and invasion assays. *p < 0.05 compared to control group; *^#^p* < 0.05 compared to the ugonin V-treated group. WT, wildtype; MUT, mutant; 3'-UTR, three prime untranslated regions; NC, negative control.

Bioinformatic analysis using the website TargetScan predicted the binding site of miR-4799-5p on CTSV 3'-UTR (Fig. 5A). We then generated CTSV wildtype (WT) and mutant (MUT; replacing the binding site with three nucleotides) luciferase reporter plasmids. Upon treatment with ugonin V, we observed a decrease in the luciferase activity of the CTSV WT reporter, while the CTSV MUT reporter showed no change (Fig. 5B), indicating that miR-4799-5p directly binds to the CTSV 3ʹ-UTR in response to ugonin V treatment.

To provide additional evidence, we pretreated chondrosarcoma cells with an inhibitor of miR-4799-5p and found that ugonin V via promoting miR-4799-5p suppresses CTSV expression. Western blotting and qPCR results in Fig. 5C, D and E show that the effect of ugonin V was rescued when we pretreated chondrosarcoma cells with the miR-4799-5p inhibitor. Migration and invasion assays revealed that ugonin V effectively inhibited the movement of chondrosarcoma cells by upregulating miR-4799-5p expression (Fig. 5F and G). Therefore, ugonin V suppressed CTSV and chondrosarcoma cell motility by promoting miR-4799-5p directed binding to the CTSV 3ʹ-UTR.

### Ugonin V inhibits chondrosarcoma metastasis *in vivo*

**Figure 6:**
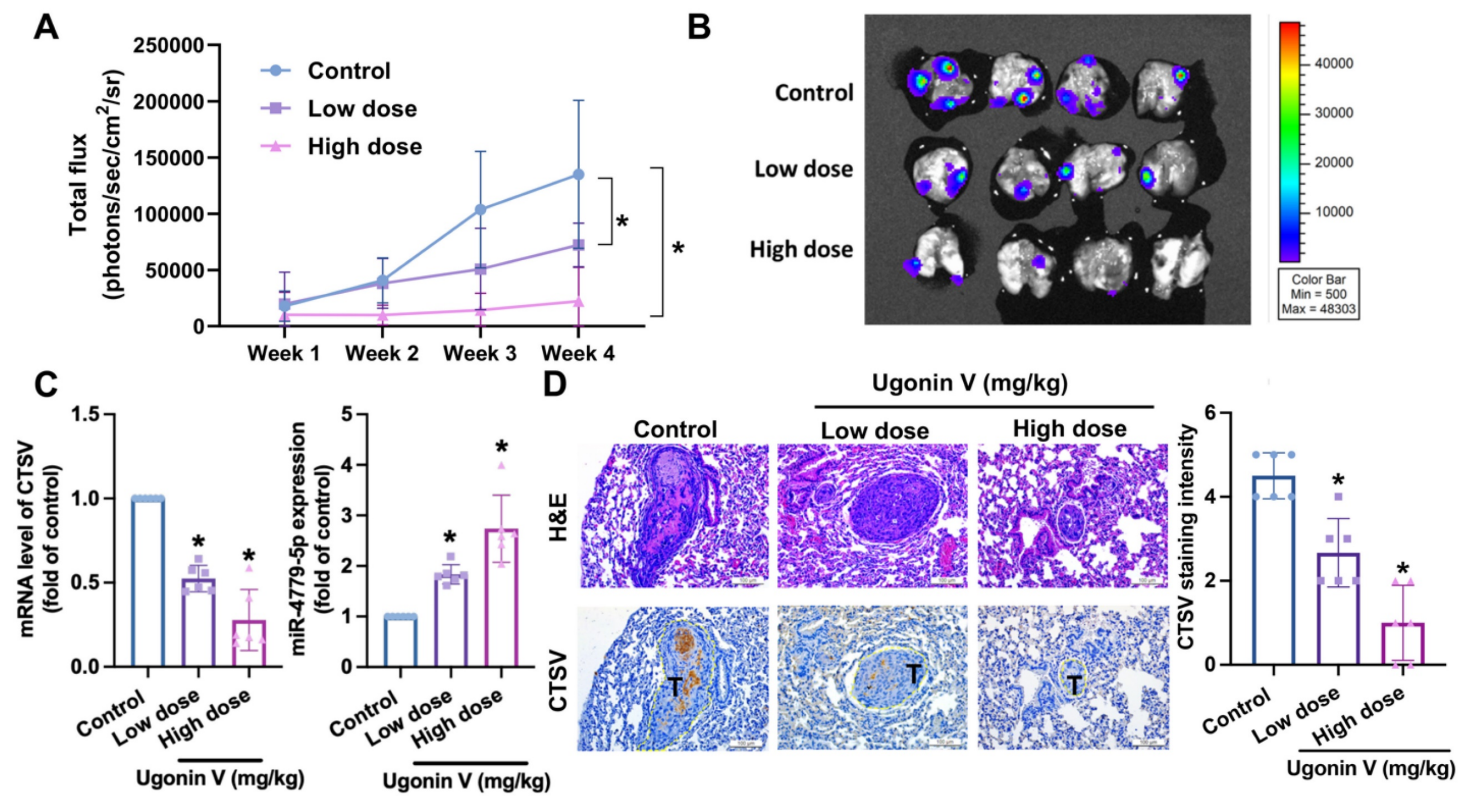
** Ugonin V inhibits chondrosarcoma metastases to the lung by modulating CTSV and miR-4799-5p synthesis.** (A) After a 4-week treatment period, all mice were euthanized and luciferase activity was measured at the indicated time using an IVIS imaging system. The bioluminescence signal intensity (photons/s/cm^2^/sr) was quantitatively analyzed*.* (B) At the end of the experiment, lung metastasis was captured and analyzed by signal intensity (photons/s/cm^2^/sr). (C) Mice's blood was collected, and miR-4799-5p expression and CTSV mRNA expression were quantified by qPCR. (D) H&E and IHC staining were utilized to identify the expression of CTSV in chondrosarcoma tumors in mice's lungs. *p < 0.05. T, tumor; H&E, hematoxylin-eosin stain; IHC, immunohistochemistry.

A chondrosarcoma lung metastasis model was established to investigate the *in vivo* effects of ugonin V. Fig. 6A and B show that the development of chondrosarcoma tumors in lungs of mice was significantly restricted in the ugonin V-treated groups during the four weeks of treatment. Blood samples were collected for qPCR analysis to detect CTSV mRNA and miR-4799-5p expression. The groups treated with ugonin V showed a notable decrease in CTSV mRNA levels and a significant increase in miR-4799-5p expression (Fig. 6C). Lung sections from mice were used for IHC staining. The results in Fig. 6D showed a remarkable reduction of CTSV in both the low- and high-dose treatment groups compared with the control group. These data demonstrate the suppressive effect of ugonin V on chondrosarcomas *in vivo*.

## Discussion

Over 50% of advanced-stage chondrosarcomas exhibit metastasis to other organs, particularly the lungs 5. In addition, late-stage chondrosarcoma is associated with poor prognosis and low survival rates 38, 39. Therefore, metastasis is a major challenge in the treatment of chondrosarcoma. Numerous efforts have been made to explore novel therapeutic approaches, including targeted therapies, for advanced chondrosarcoma. For instance, IPI-926, an inhibitor of the Hedgehog pathway, has been approved for phase II clinical trials. However, the outcomes of this trial showed no benefits in patients with advanced chondrosarcoma patients 40. Recently, ivosidenib (a mutant IDH1 inhibitor) was approved for phase I clinical trials and showed no toxicity in chondrosarcomas 41. However, to date, no targeted inhibitor for chondrosarcoma has been approved by the FDA. Therefore, targeted treatment is still a field that needs more attention in chondrosarcoma research.

The cathepsin family has been described in many studies for its essential role in cancer metastasis 42. For example, cathepsin K expression has been reported to be a predictor of the prognosis of patients with late-stage osteosarcoma 43. Overexpression of cathepsin B enhances hepatocellular carcinoma cell migration and is associated with poor prognosis patient 44. CTSV has an important role in inducing cancer cell migration in breast, lung, and bladder cancers 22, 45, 46. To date, research on CTSV and chondrosarcoma is scarce. To the best of our knowledge, this is the first study to investigate CTSV expression in chondrosarcomas. We provided evidence for CTSV detection using bioinformatics analysis, qPCR analysis, and IHC staining (Fig. 2A, C, D and E) to indicate the remarkably higher expression of CTSV in chondrosarcomas. Treatment of JJ012 and SW1353 cells with ugonin V resulted in a significant, concentration-dependent decrease in CTSV levels (Fig. 2F, G and H).

Many researchers have recently succeeded in using miRNA-based cancer treatment techniques to directly target genes known to induce tumor proliferation, angiogenesis, or metastasis 15, 47. Several studies have shown that natural products can modulate miRNA activity to hinder cancer progression 48, 49. In chondrosarcomas, the production of miR-423-5p and miR-520f-3p inhibits cell migration *in vitro* and *in vivo* 29, 50. Our study identified several novel candidates for chondrosarcoma research, including miR-892a, 892b, 377-5p, and 4799-5p. Previous studies have shown that these miRNAs act as tumor suppressors 51-53; however, there have been relatively few studies on the function of miR-4799-5p.

In particular, miR-4799-5p has been recognized as a potential biomarker for polycystic ovarian syndrome and is anticipated to target NAMPT and MAPK1, highlighting its putative involvement in the molecular pathways associated with the condition 54. Furthermore, miR-4799-5p was significantly enriched with amelogenesis imperfecta-associated genes 55. Nevertheless, fewer research examine this miRNA in relation to cancer. We first indicated that the expression of miR-4799-5p was lower in patients with chondrosarcoma (Fig. 4F). Moreover, miR-4799-5p was demonstrated to directly bind to CTSV 3'-UTR to suppress CTSV synthesis and then inhibit chondrosarcoma cell migration and invasion in ugonin V treatment (Fig. 5). In the chondrosarcoma lung metastasis model, miR-4799-5p expression in the ugonin V-treated group was significantly higher than that in the control group (Fig. 6C). Therefore, miR-4799-5p acts as a mediator of the ugonin V-induced suppression of CTSV expression and chondrosarcoma metastasis. In addition, whether ugonin V induces stimulation of another miRNA target, CTSV, and chondrosarcoma cell migration and invasion remain to be determined by further research. The present study acknowledges certain limitations concerning the miR-4799-5p mimic and its impact on downstream functions in the metastasis of chondrosarcoma cells. Furthermore, additional research is essential to elucidate the role of miR-4799-5p in animal models and clinical trials.

Natural products and their structural analogs play a significant role in pharmacotherapy, particularly in cancer treatment 56, 57. Natural extracts such as apigenin, crocin, wogonin, and curcumin have been shown to suppress cancer metastasis 58. Some ugonin compounds, belonging to the flavonoid group, such as ugonin P, J, and K, have shown inhibitory effects on cancer cell movement 15, 59. Our previous study investigated the suppressive effects of ugonin V on chondrosarcoma metastasis by targeting MMP7 expression 17. In the current study, we identified another target of ugonin V, CTSV, which is regulated by miR-4799-5p. Some flavonoids were reported to target more than one protein to inhibit cancer progression. For instance, apigenin targets IL-6 and thromboxane A2 to suppress breast cancer cell metastasis 60, 61. Another flavonoid, luteolin, inhibits lung cancer metastasis by decreasing TWIST1 and MMP2 expression 62. This study provides evidence demonstrating the multitargeted activity of ugonin V in chondrosarcoma metastasis treatment. Whether it can be applied to clinical treatments remains to be determined by further examination.

## Conclusion

**Figure 7:**
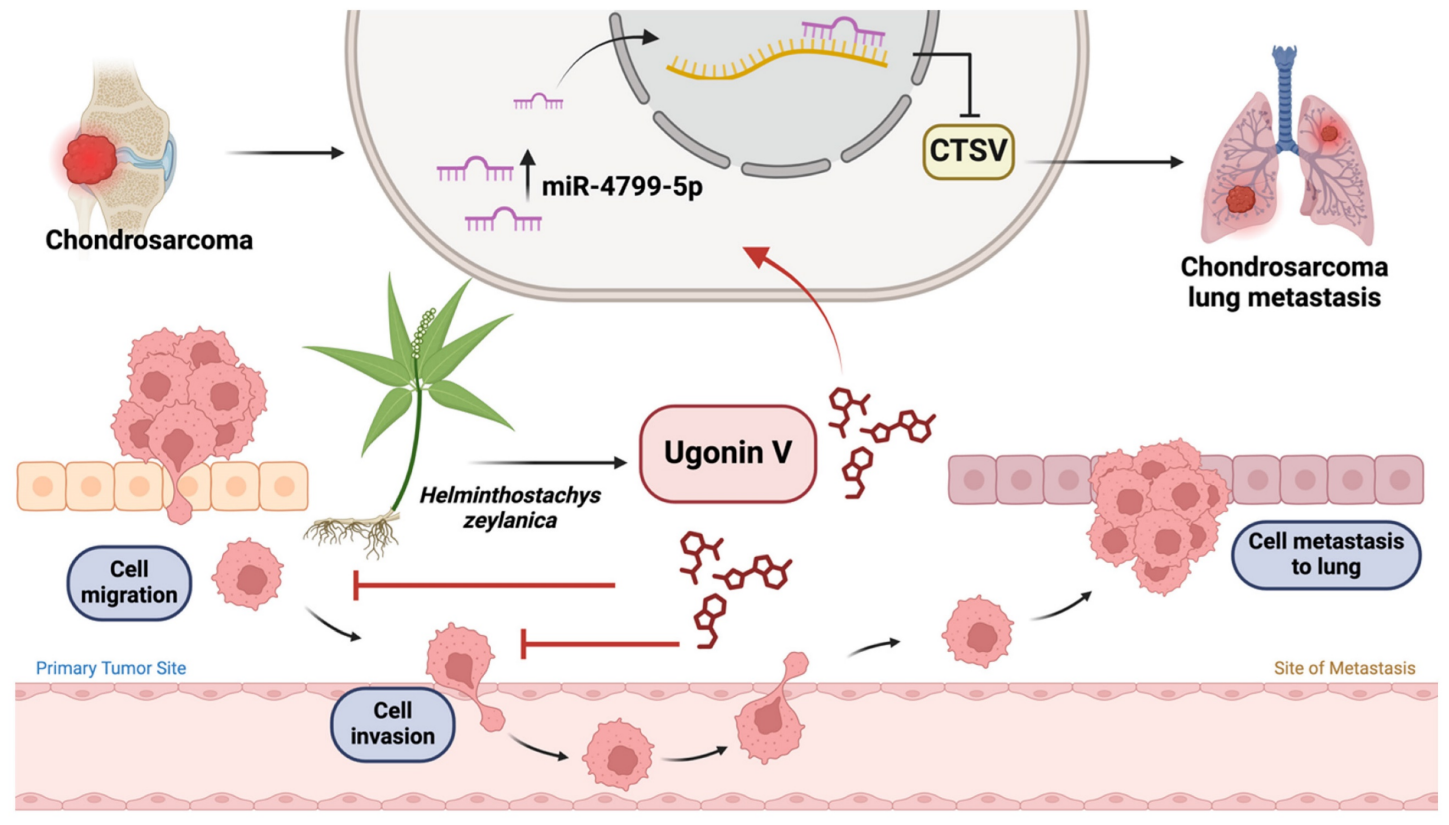
** Illustration depicting the effects of ugonin V on chondrosarcoma cell metastasis.** (The schema was generated utilizing BioRender.com). Ugonin V-induced chondrosarcoma metastasis suppression by activation of miR-4799-5p reduces levels of CTSV expression.

In conclusion, this study explored the molecular mechanisms underlying the inhibitory effects of ugonin V on chondrosarcoma metastasis. We provided *in vitro* and *in vivo* evidence for the suppressive effect of ugonin V on chondrosarcoma cell motility and metastasis via the stimulation of miR-4799-5p expression, which reduces CTSV synthesis (Fig. 7). We expect that our study identified ugonin V as a potential candidate for the treatment of chondrosarcoma metastasis.
